# Supplementary material for: Getting More Out of Biomedical Documents with GATE's Full Lifecycle Open Source Text Analytics
Source: PLoS Comput Biol. 2013 Feb 7;9(2):e1002854. doi: 10.1371/journal.pcbi.1002854 (PMC3567135; doi:10.1371/journal.pcbi.1002854)
Supplement: Dataset S1 — GATE software. Dataset S1 bundled with this paper contains a distribution of GATE (or see http://gate.ac.uk/download/). (TGZ) [file pcbi.1002854.s001.tgz › plos-gate/doc/announce-6-0.html]

GATE Version 6.0 release (November 2010)


# GATE Version 6.0 release (November 2010)

GATE version 6.0 adds a number of new features to GATE including:

- A tool to support the annotation of relational information in documents using
  an ontology.
- A *scriptable controller* to execute PRs under the control of a Groovy
  script, providing more powerful conditional behaviour than is possible with
  the standard conditional controllers.
- A new version of the alignment editor for handling aligned parallel texts
  (for example in different languages).
- A plugin to support on-the-fly translation of documents using the Google
  translation web service.
- Enhancements to JAPE, an improved gazetteer editor, integration of the NLM's
  MetaMap tagger for biomedical terms, and much more...

Full details of the changes can be found in the
changelog in the user guide.
GATE is available for download from http://gate.ac.uk/download/.
